# Transcriptional Analysis of Aggressiveness and Heterogeneity across Grades of Astrocytomas

**DOI:** 10.1371/journal.pone.0076694

**Published:** 2013-10-11

**Authors:** Chunjing Wang, Cory C. Funk, James A. Eddy, Nathan D. Price

**Affiliations:** 1 Institute for Systems Biology, Seattle, Washington, United States of America; 2 Department of Chemical and Biomolecular Engineering, University of Illinois, Urbana, Illinois, United States of America; 3 Department of Bioengineering, University of Illinois, Urbana, Illinois, United States of America; Instituto de Investigación Sanitaria INCLIVA, Spain

## Abstract

Astrocytoma is the most common glioma, accounting for half of all primary brain and spinal cord tumors. Late detection and the aggressive nature of high-grade astrocytomas contribute to high mortality rates. Though many studies identify candidate biomarkers using high-throughput transcriptomic profiling to stratify grades and subtypes, few have resulted in clinically actionable results. This shortcoming can be attributed, in part, to pronounced lab effects that reduce signature robustness and varied individual gene expression among patients with the same tumor. We addressed these issues by uniformly preprocessing publicly available transcriptomic data, comprising 306 tumor samples from three astrocytoma grades (Grade 2, 3, and 4) and 30 non-tumor samples (normal brain as control tissues). Utilizing Differential Rank Conservation (DIRAC), a network-based classification approach, we examined the global and individual patterns of network regulation across tumor grades. Additionally, we applied gene-based approaches to identify genes whose expression changed consistently with increasing tumor grade and evaluated their robustness across multiple studies using statistical sampling. Applying DIRAC, we observed a global trend of greater network dysregulation with increasing tumor aggressiveness. Individual networks displaying greater differences in regulation between adjacent grades play well-known roles in calcium/PKC, EGF, and transcription signaling. Interestingly, many of the 90 individual genes found to monotonically increase or decrease with astrocytoma grade are implicated in cancer-affected processes such as calcium signaling, mitochondrial metabolism, and apoptosis. The fact that specific genes monotonically increase or decrease with increasing astrocytoma grade may reflect shared oncogenic mechanisms among phenotypically similar tumors. This work presents statistically significant results that enable better characterization of different human astrocytoma grades and hopefully can contribute towards improvements in diagnosis and therapy choices. Our results also identify a number of testable hypotheses relating to astrocytoma etiology that may prove helpful in developing much-needed biomarkers for earlier disease detection.

## Introduction

Primary brain tumors comprise less than 2% of all human cancers but have strikingly high mortality rates. Glioma, the most prevalent primary brain tumor, accounts for ∼42% of all adult brain tumors [1]. The most common gliomas, in turn, are astrocytomas, believed to originate from astrocytes [2], [3]. Astrocytomas are classified from Grade 1 (least aggressive) to Grade 4 (most aggressive) based on the World Health Organization (WHO) grading system [4].

**Figure 1:**
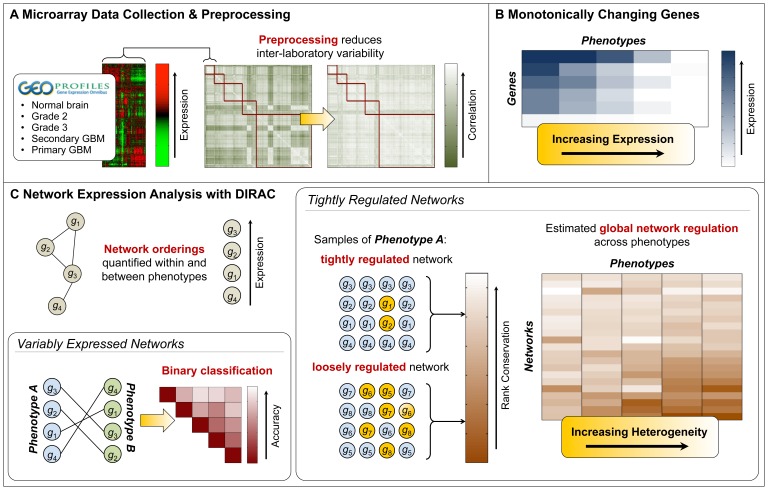
Overview of approach. **A)** We minimized experimental variation due to lab effects by performing uniform pre-processing. **B)** Genes that either monotonically increased or decreased in parallel with increasing astrocytoma grade were identified. **C)** Molecular signatures that can accurately distinguish between different grades were established using Differential Rank Conservation (DIRAC). We also examined broad patterns of network regulation across all astrocytoma grades.

We present an analysis of the different grades of astrocytoma (excluding pilocytic astrocytoma, with normal brain tissues taken as control) to identify both distinct and common molecular states across grades. We have employed a combination of gene- and network-based approaches (**Figure 1**) to investigate the genetic and biological mechanisms implicated in observed phenotypic differences. Grade 1 tumors (pilocytic astrocytomas) represent distinct pathological and biological entities compared with other tumors [5] and thus were not included in this study. As such, we will henceforth consider only Grades 2 through 4. Grade 2 (G2) and Grade 3 (G3) tend to progress to higher grades with recurrence. Grade 4 tumors (glioblastoma multiforme or GBM) commonly present as *primary* tumors (pGBM), with no prior history of occurrence at a lower grade. *Secondary* GBM (sGBM), on the other hand, has recurred in a patient previously diagnosed and treated for a lower grade [6]. Specific avenues of progression, where astrocytoma manifests in G2 tumors that undergo transformation to the more aggressive G3 or GBM tumors, have been seen in both genetically engineered mouse models [7], as well as in humans [6]. Our study included only GBMs with clear subtype designations (primary or secondary) and investigated differences between GBMs and lower grades as well as between these subtypes.

Many studies have aimed to classify or stratify astrocytomas using genomic, transcriptomic, and integrated approaches with varying success. Subtypes of GBMs with common *molecular* characteristics (i.e., as opposed to clinical presentation) have been identified [8]. While there is continued hope that identification of these subtypes will lead to clinically distinct treatments, to date, no such treatments have emerged. Identification of these subtypes has, however, aided in our understanding in several of the molecular drivers of astrocytoma. One recent report identified IDH mutation in Grades 2 and 3 astrocytomas as an indicator of better clinical outcome–addressing a pertinent challenge, as these lower-grade astrocytomas can have widely variable outcomes [9].

While genomic studies have been able to identify several common and informative mutations, transcriptomic studies have not proven as robust. Differentially expressed genes and molecular signatures have been identified in previous microarray experiments, in an attempt to address clinical needs [10]–[15]. Unfortunately, as most of these studies were statistically underpowered, these signatures failed on independent validation sets, thus rendering them ineffectual [16]. Lab effects can obfuscate signal from noise in phenotypically similar tumors, if sampled from different studies [17]. This can be overcome through use of multiple datasets when properly normalized–also minimizing the inherent biological noise [18]. Our present study adopted such a uniform approach to process raw expression data from multiple labs with one standard adjustment method, thereby increasing sample-to-sample correlation and decreasing heterogeneity across the data collected in different studies (**Figure 1A**).

Another strategy to mitigate biological noise is to analyze molecular profiles from individual genes or proteins in the context of biological network behaviors–such a strategy also helps to link changes in gene expression to phenotype. Studying network behavior is especially relevant in cancer research, as cancer stages and progression are marked by changes in network-level processes [19].

We previously developed a method called Differential Rank Conservation (DIRAC) [20], which measures the variation in *network ranking* (i.e., the relative ordering of genes from highest to lowest expression within a pre-defined network) among samples of the same phenotype and between samples of different phenotypes (**Figure 1C**). This enables evaluation of changes in gene expression at a network level based on relative expression between each of the network components, making the method independent of any normalization that does not affect rank (e.g., normalizing to total RNA, quantile normalization, etc.); additionally, the results do not depend on the other genes in the transcriptome, meaning that it can be applied when only the genes in the selected network are measured. We first examined broad patterns of network regulation across all astrocytoma grades using DIRAC and observed a trend of greater network dysregulation by increased sample-to-sample variation of gene ordering within networks with increasing astrocytoma grade. We also identified the most differentially regulated networks between different grades (i.e., heterogeneous in one grade but significantly less so in another).

This tendency of increased transcriptomic heterogeneity at both the global- and individual-network level for more aggressive astrocytomas led us to explore potential underlying patterns of expression among *individual genes*. We thus identified genes that either monotonically increased or decreased in parallel with increasing astrocytoma grade (**Figure 1B**); furthermore, we observed numerous associations between these genes and functional categories implicated in cancer including calcium signaling, mitochondrial metabolism, and apoptosis. Given the significant genomic heterogeneity found in cancer, that specific genes are monotonically changing in expression with much more prevalence than would be expected by chance may be reflective of shared oncogenic mechanisms among phenotypically similar tumors.

Amidst the heterogeneous expression of higher grades of astrocytoma, we identified networks that exhibit *consistent changes* between phenotypes, establishing molecular signatures that can potentially offer mechanistic explanations for differences between grades. DIRAC provides an informative metric for network-based classification that may be more capable of extracting sufficient signal from noise than gene-based approaches. While it was difficult to reliably distinguish sGBMs from lower grades, as expected, we identified more robust signatures separating pGBMs from other grades (**Figure 1C**). Investigating these networks in greater depth provides a starting point for characterizing underlying mechanisms for aggression in astrocytomas.

## Methods

### Collection and Integration of Transcriptomic Data

Raw microarray CEL files from previous studies were compiled from the NCBI Gene Expression Omnibus (GEO). We used data collected from the most abundant source platform currently, Affymetrix HG-U133A or its complimentary version, HG-U133-Plus 2.0 GeneChips (Affymetrix, Santa Clara, CA). **Table 1** lists the GEO accession number, year of publication, and the number and grades of samples reported in each original study.

**Table 1:** Summary of microarray expression datasets included in the study.

Platform	Authors of Study (year, GSE accession)	Number of patients in each class				
		Normal	Grade 2	Grade 3	pGBM	sGBM
U133A	Freije *et al* (2006, GSE 4412) [12]	0	0	8	34	12
	Phillips *et al* (2006, GSE 4271) [13]	0	0	21	55	0
	Wong *et al* (2008, GSE12907) [14]	4^a^	0	0	0	0
	Rich *et al* (2005, GSE 13041) [62]	0	0	0	31	0
	Lee *et al* (2008, GSE 13041) [63]	0	0	0	15	13
	Barrow *et al* (2008, GSE 13041) [63]	0	0	0	28	3
	Mcdonald *et al* (2005, GSE 3185)	0	3	0	0	0
Total U133A		4	3	29	163	28
U133-Plus 2.0	Sun *et al* (2006, GSE 4290) [15]	23^b^	7	19	0	0
	Liu *et al* (2010, GSE 19728)	1^c^	5	5	0	0
	Lee *et al* (2008, GSE 13041) [63]	0	0	0	11	16
	Turkheimer et al (2006, GSE 2817) [64]	0	6	0	0	0
	Chow et al (2010, GSE 22927) [65]	0	6	0	0	0
	Grzmil et al (2011, GSE15824) [66]	2	4	4	0	0
Total U133-Plus 2.0		26	28	28	11	44
**Total**		**30**	**31**	**57**	**174**	**44**

a Includes one normal fetal brain RNA, one normal cerebellum RNA, and two normal tissues surgically removed tissue adjacent to resected tumor tissue and RNA extracted.

b Brain samples of epilepsy patients.

c Pooled normal brain tissue.

A “consensus pre-processing” method was applied to the CEL files to normalize differences introduced by non-uniform studies and sample preparation procedures. This method is described in greater detail in [18] and was used in that study to demonstrate that classifiers performed better on novel datasets when trained on multiple, integrated, pre-processed datasets. Briefly, common probe sets (22,277) shared by the two platforms (U133A and U133-Plus 2.0) were identified according to Affymetrix descriptions, and GeneChip RMA (GC-RMA) normalization was applied to raw expression data for these probes across all microarray samples [21]. GC-RMA was implemented in the Matlab Bioinformatics Toolbox with the threshold for presence defined based on prior studies from Affymetrix [22]. Probes having 0% present calls for any phenotype were removed. Following these criteria, 15,827 probes were kept for further analysis.

When converting the probe intensity matrix to a gene expression matrix, probes that mapped to multiple genes were eliminated to remove ambiguity. For multiple probes corresponding to the same gene, the maximum intensity was used. Finally, all absolute intensity values were replaced by their relative ranks within each array.

### Computation of Rank Conservation Indices in DIRAC

For all network analyses performed with DIRAC, expression levels of genes were grouped into 248 human signaling networks, defined according to the BioCarta gene sets collection in the Molecular Signatures Database (MSigDB) [23]. For each selected network, we used DIRAC to compute the expected ordering of network genes (rank template) for each phenotype, and we subsequently measured how closely each sample’s network ordering matched the phenotype-specific template (rank matching score). The rank conservation index, calculated by averaging rank matching scores across samples in a phenotype, indicates how consistently each network is ordered within a population. Averaging the rank conservation indices over all networks for a phenotype provided a single value estimating the relative heterogeneity or *dysregulation* of networks for that phenotype.

### Identification of Most Differentially Regulated Networks Across Grades

The difference in rank conservation indices between two phenotypes (e.g., normal vs. cancer or lower grade vs. higher grade) was calculated for each network. Networks were ranked based on the magnitude of the difference. To establish statistical significance, the original phenotype labels were permuted and randomly assigned to samples, and the absolute difference in rank conservation indices was calculated for all networks in each phenotype. These steps were repeated for 1,000 permutations to generate a null distribution of rank conservation differences, and the significance level for each difference was measured as the probability of observing the same fraction or higher at random.

### Identification of Monotonically Changing Genes

We selected differentially expressed genes (DEGs) for each adjacent pair of astrocytoma grades, (control vs. G2, G2 vs. G3, etc.) based on the Wilcoxon rank-sum test (*P*<0.05 after Bonferroni correction). In the intersection of these DEG sets, genes with monotonically increasing ranks were defined as *increasing* genes, and monotonically decreasing ranks as *decreasing* genes.

In order to test the robustness of these monotonically changing genes, we randomly selected 80% of all samples in each phenotype, and with this subset of samples, we tracked whether genes were similarly increasing or decreasing across grades as they were with the full set of samples. We repeated this selection process 1,000 times and recorded how often the identified genes appear with the same pattern. Genes that appeared at least 500 times in 1,000 permutation tests were considered as high-confidence genes and used for subsequent analysis.

In order to test the significance of the directionality of genes, the original phenotype labels were permuted and randomly assigned to samples, and the number of monotonically changing genes in each permutation was calculated for both the increasing and decreasing case. The procedure was repeated for 1,000 times and a null distribution for the gradation of genes was established. A *P*-value for the directionality/trend of the genes was assigned based on the probability of observing the same number of genes at random.

### Classification of Disease Phenotypes with DIRAC

In addition to conservation of network ordering within a phenotype (measured by the rank conservation index), DIRAC can also be used to identify networks ordered differently (variably expressed) between two phenotypes. Rank matching scores were calculated for each class, and predicted class labels were assigned based on similarity of each patient’s individual profile to either of the two templates. Apparent accuracy for classification with these predicted class labels was then calculated for all networks [20]. A null distribution of network classification rates was generated by randomly permuting phenotype labels 1,000 times, and the significance level was measured as the probability of observing classification rates. To address the issue of multiple-hypothesis testing, the corresponding false discovery rate (FDR) was calculated for each significance level, representing the fraction of expected false positives at any defined cutoff [20]. We used leave-one-out cross validation to estimate the error rate of DIRAC-based classification for each pair of phenotypes.

## Results and Discussion

### Consensus Pre-processing Reduces Noise and Increases Homogeneity Across Microarray Datasets

Appropriate computational pre-processing is an important step in combined analyses of multi-site data to reduce technical variability between different studies. Consensus pre-processing, which normalizes raw expression data from multiple studies in a uniform manner, has been shown to reduce lab effects; such lab effects are known to obfuscate biological signal when combining datasets from multiple labs [18]. Molecular signatures obtained after this step of processing have better prediction accuracy and lower variance than those from individual datasets. For example, average accuracy obtained training on four GBM datasets was considerably higher than training on individual GBM datasets [18]. We applied consensus pre-processing to the raw expression data for 336 patients collected from multiple independent studies. This greatly reduced sources of variation across studies, as measured by an increase in average sample-to-sample correlation from 81% to 91% (**Figure** S**1**). Reducing noise in the data enabled a more robust identification of variability across phenotypes.

### Global Dysregulation of Networks Across Astrocytoma Grades Coincides with Aggressiveness

We first investigated global differences in network-level expression between astrocytoma grades by applying DIRAC to measure the rank conservation index of relative stability or consistency within each network ordering across a population [20]. If the orderings of genes within a specific network are mostly similar among different patients (i.e., highly conserved), the network is considered *consistent* within a phenotype. In the opposite case, more dissimilarity among patients is observed, and the network is considered *heterogeneous* or *dysregulated.* Extending this concept, averaging rank conservation indices over all networks provides a coarse measure of global regulation in different phenotypes.

**Figure 2:**
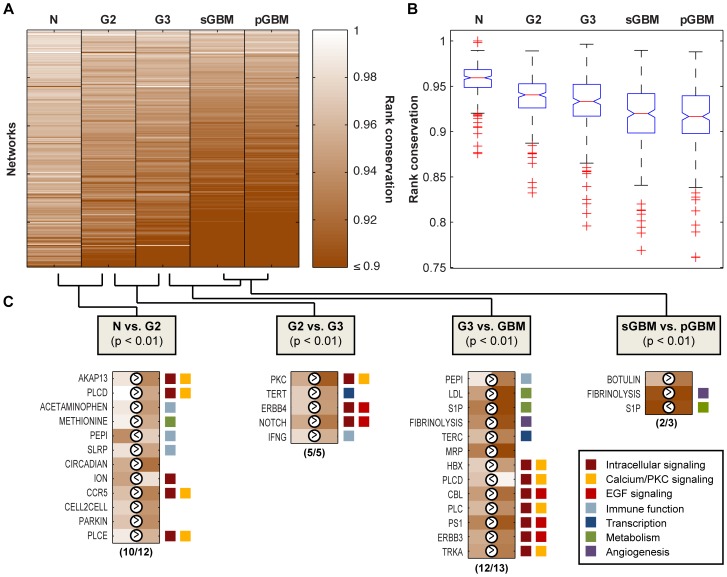
Network-level expression heterogeneity across tumor grades. **A)** Global trend of network regulation level decreases with increeasing grade. The vertical axis represents examined networks, while the horizontal axis represents five phenotypes. Colors represent rank conservation indices for each network. Light colors indicate high consistency of network ranking in a phenotype and the dark colors indicate large heterogeneity of networks. Networks in sGBM and pGBM tumors become much more heterogeneous compared to the normal cases. **B)** One-way ANOVA comparing the mean rank conservation values of different phenotypes. **C)** A list of most deregulated networks between adjacent tumor grades and their major biological functions. The “>” and “<” indicate the magnitude of network regulation. For instance, AKAP13 has a larger rank conservation index in normal samples and thus is more regulated in normal compared to G2 patients.

We found that networks in normal brains are on average more highly conserved (0.957) than networks in advanced astrocytoma grades (G2, 0.937; G3, 0.930; and GBM (including both pGBM and sGBM), 0.915; *P*<0.001 for ordering of phenotypes, based on one-way ANOVA) (**Figure 2A&2B**). In addition, global network rank conservation is significantly different between all pairs of phenotypes (*P*<0.05, multiple pair-wise t-tests). This trend demonstrates that more aggressive phenotypes have greater overall variation in network ordering among different samples. Increased genetic and cellular heterogeneity is a commonly recognized characteristic of highly malignant astrocytomas [24], [25]. GBM, the most malignant grade, is characterized by extensive heterogeneity as reflected in the moniker “multiforme”, which derives from early histopathologic descriptions of a single tumor’s highly varied morphologic features and connotes cellular heterogeneity [26]. Here, we show in a quantitative manner that *transcriptomic heterogeneity*, observed at the population scale, is generally correlated with increasingly aggressive phenotypes.

### Differentially Regulated Networks between Disease States Correlate with Gliomagenesis and Development

Certain networks appear consistent in one phenotype but show drastically more sample-to-sample heterogeneity in another phenotype. Identifying the most differentially regulated networks can inform us about cellular processes and mechanisms most affected or perturbed from one disease state to another. We thus identified the most differentially regulated networks between normal samples and each astrocytoma grade as well as between different disease states (**Table S1**). For example, we identified 12 out of 248 networks that had a significant difference in conservation when comparing normal to G2 patients (*P*<0.01 for each comparison, based on a binomial distribution; see **Methods**); 10 out of these 12 networks showed increased dysregulation in G2. Similarly, when comparing G2 to G3 and G3 to primary or secondary GBM, a strong majority of significantly dysregulated networks exhibited greater heterogeneity in the more malignant phenotype (**Figure 2**) (*P*<0.01 for each comparison, based on a binomial distribution). These quantitative results further support the idea that networks become increasingly dysregulated with increased malignancy.

#### Dysregulated networks in G2 vs. normal are heavily involved in intracellular calcium signaling, PKC signaling, and immune activities

Among the 12 significantly differentially regulated networks (*P*<0.01) between G2 and normal brain, 5 networks (**PLCD**, **PLCE**, **AKAP13**, **CCR5**, and **ION)** are known to regulate protein kinase C (PKC) signaling and increase calcium release into the cell (**Figure 2C**). Calcium signaling is a key player in neuronal transmission, microglia activation, and motility. Calcium signaling is especially crucial for transformed glioma cells to expand in the early stages of tumor development by sheer motility, as glioma cells cannot spread through the bloodstream [27]. Similarly, hyperactive PKC signaling is among the most distinguishing features of malignant brain tumors. PKC signaling stimulates both MAPK/ERK and PI3K/AKT pathways; it also supports degradation of extracellular matrices and allows for invasion of glioma cells [19].

Three networks, **ACETAMINOPHEN**, **SLRP**, and **PEPI**, mediating immune system responses, also showed increased dysregulation in G2 patients (**Figure 2C**). The **ACETAMINOPHEN** network was named after the commonly used drug Acetaminophen to reduce pain, targeting the cyclooxygenase enzymes. This network is also involved in inducing expansion of myeloid-derived suppressor cells (MDSC), which suppress T-cell responses to tumor growth [28]. Increased instability of this network may contribute to gliomagenesis by supporting development of MDSCs and their accumulation in the tumor microenvironment [29]. The **SLRP** network consists of 5 small leucine-rich proteoglycans (SLRPs), which are ligands of the Toll-like receptors responsible for regulating innate inflammatory responses [30].

In contrast to the **ACETAMINOPHEN** and **SLRP** networks, **PEPI** showed significantly more consistent expression ordering in the cancer population (0.877 in normal and 0.945 in G2 patients, *P* = 0.006). This network activates neutrophils and generates the wound cleaning response–and is likely indicative of the normal physiological response to most tumors. In the early stage of forming malignant glioma cells, it is possible that some immune-related networks like **PEPI** act to prevent tumor cell migration and invasion through a more consistent expression program, while the dysregulation of other networks like **SLRP** and **ACETAMINOPHEN** contributes to the immunosuppressive environment in the tumor.

#### Dysregulated networks in G3 vs. G2 induce EGFR/ErbB signaling and telomerase activation

Comparing network states in high-grade G3 to low-grade G2, all 5 networks with a significant change in consistency of gene ordering showed greater heterogeneity in the more aggressive cancer grade (**Figure 2C**). **ERBB4** and **NOTCH** networks are part of the larger EGFR/ErbB signaling pathway. The key components in this pathway consist of four members of ErbB family of proteins (Erb1-4), which tend to form heterodimers and bind several cognate growth factors (e.g., EGF, TGF), activating downstream transcription factors (e.g., JUN, FOS) to regulate multiple cellular responses including proliferation and apoptosis [31]. This pathway has demonstrated substantial biological and transcriptional consequences such as activating downstream PI3K/AKT, PKC, and MAPK/ERK pathways. Up to 40% of GBMs display deletions in EGFR rendering it constitutively active, while others overexpress it through amplification or up-regulation of expression [32].

The **NOTCH** network interacts closely with EGFR to facilitate tumor angiogenesis. Our observation that **NOTCH** shows greater variability in expression ordering at the higher grade–from 0.908 (in G2 tumors) to 0.856 (in G3 tumors)–supports the hypothesis that it plays different roles in tumorigenesis of low-grade astrocytomas and high-grade gliomas. That is, while inactive **NOTCH** functions as a tumor suppressor in low-grade G2 tumors, it is activated and may act as an oncogene in high-grade astrocytomas, especially primary GBM [33].

The **TERT** network, responsible for telomerase activation, also showed greater dysregulation in G3 compared to G2. Telomerase activation and subsequent telomere maintenance are generally associated with the malignant transformation of normal cells to cancer cells [34]. The increased transcriptomic heterogeneity and network ordering inconsistency in higher-grade astrocytomas further supports the known fact of telomerase dysregulation in malignant cancer phenotypes [35].

#### Dysregulated networks in GBM vs. G3 represent perturbed and aberrant activities in PKC, calcium, EGFR, immune system, and metabolic signaling

We compared network conservation values between G3 and primary GBM and between G3 and secondary GBM separately, and obtained 38 and 16 differentially regulated networks, respectively. 13 networks appeared as significant in both comparisons (*P*<0.01). GBM displays all the pathological features in the lower grades such as altered regulation in transcription and metabolism, calcium, and EGFR signaling (**Figure 2C**).

The **PLCD**, **PLC**, **TRKA**, and **HBX** networks all regulate release of intracellular calcium and function in similar ways as **PLCE** and **PKC**, identified in the lower grades. Notably, **HBX** includes 4 genes (GRB2, HRAS, SHC1, SOS1) that are part of the PI3K/AKT pathway–known to be hyperactivated in GBMs, resulting in uncontrolled cell growth, survival, proliferation, angiogenesis, and migration [36].

As expected, there are a number of networks involved in the complex EGFR regulatory pathway as in the other grades. The **CBL** network contains the ubiquitin ligase Cbl, which degrades EGFR and thus down-regulates EGFR signaling [31], [37]; the **ERBB3** network likewise contains functionally similar components and plays a similar role in EGFR signaling. **TERC**, another network in this list, behaves like **TERT** to control telomerase regulation.

Interestingly, two networks (**LDL** and **S1P**) with critical roles in cholesterol metabolism also displayed significant dysregulation in GBM. **LDL** transports cholesterol, which is needed for cell membrane repair and synthesis, whereas **S1P** controls transcriptional regulation of cholesterol metabolism in response to cholesterol levels in the cell [38]. In addition, **S1P** connects to the earlier mentioned EGFR pathway through two sterol-regulatory element-binding proteins (SREBF1 and SREBF2) that are activated by PI3K. The interplay between S1P, SREB proteins, and EGFR regulates the expression of fatty acid synthase, which synthesizes fatty acids and plays a key role in cancer pathogenesis [39]. It has been reported that EGFR mutations (EGFRVIII) and PI3K promote tumor growth and survival through SREBP-1 dependent lipogenesis [40].

#### Dysregulated network distinguishing pGBM from sGBM is related to IDH mutation

In comparing the two subtypes of GBM, primary to secondary GBM, it is interesting to note that the conservation value of **S1P** also decreased significantly from 0.812 in pGBM to 0.769 in sGBM (*P* = 0.005). The SREBF1 gene in this network regulates and activates the IDH1 gene [41]. IDH mutations are commonly observed in lower-grade and secondary GBMs but rarely in primary GBMs [42]. Thus, this network links IDH mutation to lipid homeostasis. Increased network dysregulation of **S1P** in sGBM offers quantitative support that IDH signaling is altered in this subset of GBMs.

### Monotonically Increasing and Decreasing Genes in Astrocytoma Progression

Amidst the increased dysregulation of gene networks with increasing astrocytoma grade, we sought to identify instances where specific molecular changes–in this case, changes in expression of individual genes–occur in a unidirectional manner. We reasoned that such instances could provide insight into the oncogenic mechanisms or events that contribute to the pathology and/or transcriptomic heterogeneity found in astrocytoma. We therefore looked for genes whose expression level monotonically changed concomitant with increasing grade.

**Figure 3:**
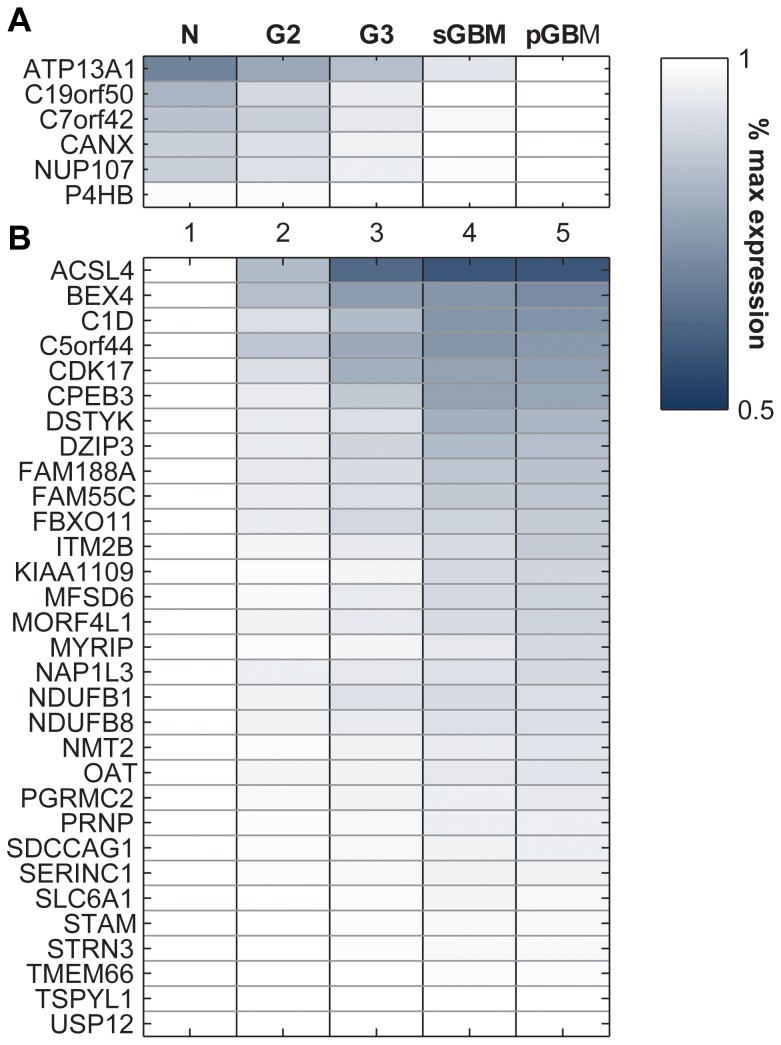
Genes showing consistent differential expression with progression. Colors on the heatmap represent relative expression values of genes in different phenotypes. The vertical axis lists the differentially expressed genes and the horizontal axis lists the phenotypes. All expression values are normalized as the percentage of maximum expression value for the gene across all phenotypes. For the up-regulated genes in panel **A)** the maximum expression is either sGBM or pGBM, so we see all genes have brightest color in these two phenotypes; similarly the down-regulated genes in panel **B)** decrease their expression systematically from normal to GBM, and the intensity level also increases with grade.

31 and 6 genes were found to decrease or increase their respective expression from normal to G2, G3, and GBM (**Figure 3** **, Table S2**). In evaluating DEGs between G3 and GBM, only genes differentially expressed in both pGBM *and* sGBM compared to G3 were included (see **Methods**). We also tested for the statistical significance of the directionality of the genes (*P*<0.001, see **Methods**). The fact that specific genes change consistently with increasing astrocytoma grade may reflect shared oncogenic mechanisms among phenotypically similar tumors. Interestingly, similar to the differentially regulated networks, several of these genes identified are also associated with key processes such as calcium signaling and metabolism and/or are located in the endoplasmic reticulum (ER) or mitochondria. The commonalities shared by gene-based and network-based analysis may represent potential connections between genetic heterogeneity at the tumor level and expression heterogeneity at the population level.

**Figure 4:**
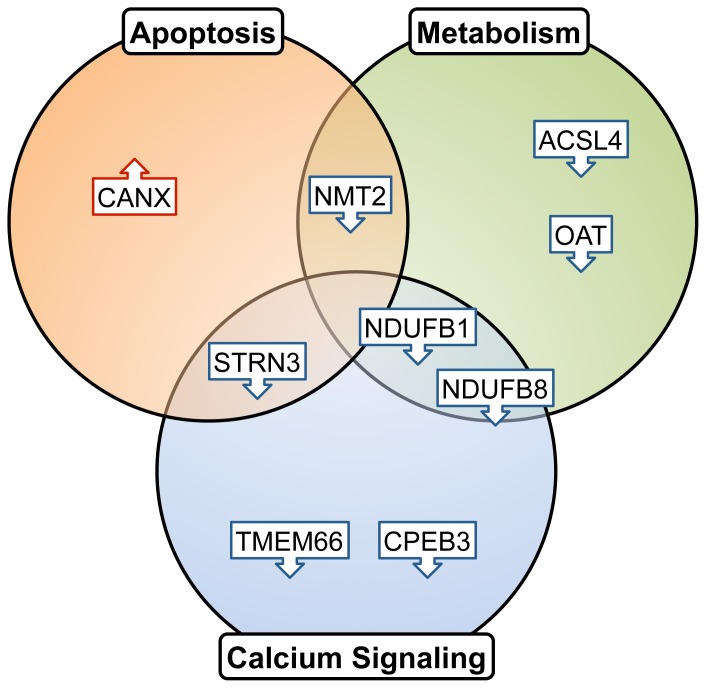
Functional categories among monotonically changing genes.

The significance of calcium signaling and metabolic genes may relate to how cells respond to additional metabolic requirements needed for tumor cell division and cell cycle progression with increased aggressiveness. At the same time, cells that ultimately constitute the tumor mass have been selected for their ability to avoid apoptosis while facilitating increased metabolic flux. As such, we see genes implicated in regulation of apoptosis. We discuss in detail below how representative genes are involved in the above-mentioned functional categories and how they interact to bring about changes reflective of astrocytoma pathology. A summary of the genes and their respective functions is shown in **Figure 4**.

#### Genes implicated in calcium signaling and/or apoptosis

Among the monotonically changing genes, TMEM66, STRN3, CANX, and CPEB3 are known to affect calcium and apoptotic signaling; all of them, with the exception of CANX, showed decreased expression with increasing tumor grade.

TMEM66, also known as SARAF, is localized to the ER lumen and affects calcium storage [43]. Following calcium release from the ER, calcium stores are replenished through calcium release activated channels (CRAC) to re-enter the ER lumen [44]. Decreased SARAF, as we observed in our study, would potentially lead to an inability to close the CRAC channels and disrupt calcium homeostasis in aggressive gliomas.

Striatin, calmodulin binding protein 3 (STRN3) is another monotonically decreasing gene and participates in apoptosis and calcium release. It is found to be both cytosolic and membrane-bound and is expressed primarily in the brain and muscle [45]. STRN3 binds with calmodulin in the presence of calcium [46]. It reacts with protein phosphatase 2a (PP2a), which, along with the promyelocytic leukemia (PML) protein, stimulates IP_3_R-mediated Ca^2+^ release from the ER. PML modulates calcium-mediated apoptotic stimuli through binding with PP2a and IP_3_R [47]. Decreased expression of STRN3 in aggressive gliomas likely reflects altered apoptotic calcium signaling mechanisms in these tumors.

Cytoplasmic polyadenylation element binding protein 3 (CPEB3) is a nucleocytoplasmic shuttling RNA-binding protein. It is involved in both calcium signaling and EGFR degradation. CPEB3 inhibits EGFR expression by preventing the translation of STAT5B, a regulator of EGFR transcription [48]. As a monotonically decreasing gene, lower expression of CPEB3 would similarly lead to an increase in EGFR. Notably, CPEB3 is located on chr10q 23.32, very close to the locus of PTEN (chr10q 23.31). Loss of this region is known to occur in several cancers [49], and it is conceivable that loss of CPEB3 contributes to altered EGFR signaling along with PTEN loss.

In contrast to the above three genes, calnexin (CANX) was found to increase at the mRNA level with increased astrocytoma grade. CANX is an ER chaperone protein that binds with free calcium ions. It is a critical component of the mitochondria associated membrane (MAM), with over 80% of it located in the MAM, along with the aforementioned STRN3-associated protein PP2A. CANX regulates the activity of sarcoplasmic/endoplasmic reticulum calcium ATPase (SERCA) by acting as a calcium buffer in the MAM [50]. Depending on its palmitoylation status, CANX shuttles between the ER and MAM [51].

#### Genes implicated in metabolism and mitochondria

A few monotonically changing genes identified have metabolic functions. Proteins encoded by these genes sit closely to each other in the mitochondria, which is responsible for essential cellular processes such as energy production, storage of calcium ions, and cell death. In recent years, there has been increased reports of the role of mitochondria in calcium signaling [52], which helps to connect mitochondrial metabolic genes with calcium signaling. Metabolic regulation of calcium in mitochondria is mediated through the effects of dehydrogenases. Calcium ions activate matrix dehydrogenases, increase available NADH and electrons for the respiratory chain, and eventually accelerate ATP production [53].

NDUFB8 and NDUFB1 are two monotonically decreasing genes that encode subunits of respiratory chain NADH dehydrogenase complex I. Decreased expression of these proteins causes respiratory chain dysfunction, reducing the driving force for calcium transfer and available electrons in the respiratory chain, and thereby decreasing ATP production. This observation may reflect a reduction of mitochondrial ATP synthesis via oxidative phosphorylation–contributing to the Warburg Effect; as the tumor grows to more aggressive stages, the metabolism of proliferating tumor cells is adapted to a proliferation mechanism rather than efficient ATP production [54]. Interestingly, the NDUFB8 gene is also found very close to PTEN (locus of NDUF8: chr10q 24.31 and PTEN, chr10q 23.31).

ACSL4 (acyl-CoA synthetase), a monotonically decreasing gene, converts fatty acids to fatty esters and plays an important role in lipid metabolism. Similar to CANX, ACSL4 is also found in MAM, which is a critical metabolic hub in lipid metabolism [55]. Though normally recognized as a metabolic gene, ACSL4 regulates synaptic vesicles along axons. Knockout of ACSL4 in embryonic stem cells was shown to significantly reduce neuronal differentiation [56]. A de-differentiated neuronal state in higher-grade tumors resembles how neural stem cells display higher potential for proliferation and angiogenesis [57].

Other mitochondrial genes involved in metabolism include ornithine aminotransferase (OAT), a monotonically decreasing gene that converts arginine and ornithine into neurotransmitters glutamate and GABA. GABA receptors and glutamate transporters have been reported to be down-regulated in brain tumors [58]. Another mitochondrial gene identified is N-myristoyltransferase 2 (NMT2) which plays a role in protein myristoylation, proliferation, and apoptosis [59].

### DIRAC-based Classification Identifies Accurate Network Signatures for Distinguishing Grades

**Figure 5:**
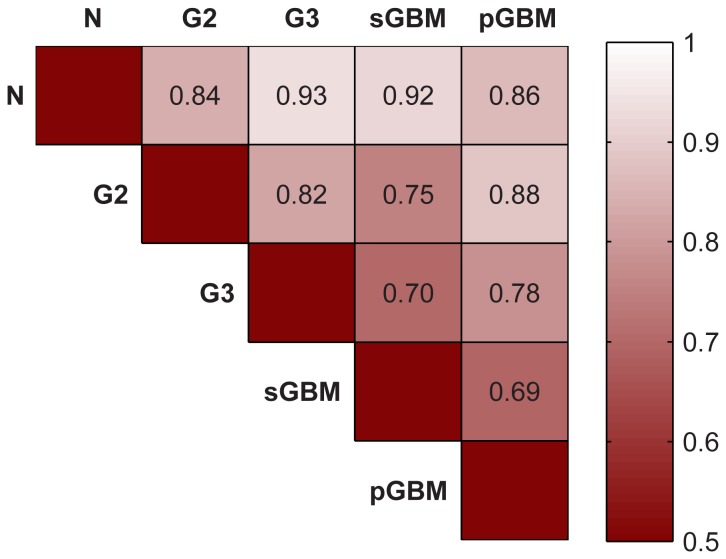
Classification accuracy with BioCarta database networks. This heatmap displays leave-one-out cross-validation accuracies of DIRAC-based classifications on each phenotype vs. all other phenotypes. DIRAC could distinguish more distant grades like normal vs. GBMs; it is especially hard to separate G3 or pGBM from sGBM.

The high degree of transcriptomic heterogeneity observed in increasingly aggressive astrocytoma tumors creates substantial variance when searching for robust molecular signatures between grades. Still, identifying such signatures is critical to elucidating mechanistic differences between more and less aggressive tumors. Network-based approaches such as DIRAC are advantageous for extracting signal from noise, as the patterns of functional groups might be less *within the same phenotype* than those of individual genes. Furthermore, DIRAC quantifies the relationships between genes, and operates on these pair-wise expression patterns within networks, thereby reducing the impact of noisy changes in single gene expression. Using DIRAC, we compared each of the four phenotypes to all other phenotypes (e.g., normal brain against G2, G3, GBM; G2 against G3 and GBM; etc.) (**Figure 5**).

We were able to clearly separate normal brain tissues from G2, G3, and primary GBM. Furthermore, these tumors could be distinguished from each other with good accuracies (>80%, except 78% in the case of G3 and pGBM). In separating secondary GBM from other grades, however, classification signals are not as strong (average accuracy of pGBM and sGBM vs. all other phenotypes are 86% and 77%, respectively). This difference in classification performance very likely reflects the fact that secondary GBMs are derived from lower grades and therefore share more common genomic and transcriptomic characteristics in their expression profiles compared to primary GBMs, which develop spontaneously and display more pronounced phenotypic differences from other grades.

It was also difficult to separate primary and secondary GBMs (accuracy 69%) based on their transcriptomes, even though they are known to develop from separate genetic pathways [60]. They are indistinguishable by histology, as both share the same histological grade [61]. Both subtypes share a number of genomic and transcriptomic similarities such as LOH on chromosome 10q and deregulation of the PI3K/ATK pathway [60]. Another reason for the relatively low accuracy is possibly due to the signal present from other subtypes such as proneural (PN), mesenchymal, or proliferative subtypes (the latter two collectively known as non-PN) within GBMs, which appear to be more distinct than the transcriptomic differences we observe between primary and secondary GBM. In terms of survival, the PN subtype is reported to be less aggressive than other subtypes [13]. In support of this hypothesis, we applied DIRAC on a subset of GBM with known PN/non-PN designations, and separated the proneural subtype from the rest with an accuracy of 78%. This accuracy being higher than for the separation of pGBM and sGBM suggests that molecular subclasses in glioblastomas may look more different than traditional pGBM/sGBM classes, especially in the context of network behavior; hence DIRAC detected the stronger classification signals more easily. The best 10 network-based classifiers selected by DIRAC to separate tumor samples from normal brains are listed in **Table 2**. In each pair-wise comparison, we included different metrics (sensitivity, specificity, and accuracy) and group size information to demonstrate the ability of DIRAC to distinguish different grades of brain tumors (**Table S3**).

**Table 2:** Top networks selected by DIRAC to classify tumor grades vs. normal brains (*P-value* <0.0001).

BioCarta Network	Apparent Accuracy	BioCarta Network	Apparent Accuracy
PTDINS	0.918	MCALPAIN	0.966
EGF	0.918	PTDINS	0.945
FAS	0.918	ERK	0.940
TNFR1	0.918	G2	0.932
RACCYCD	0.902	G1	0.932
CBL	0.902	EGF	0.932
PDGF	0.902	CELLCYCLE	0.925
IL1R	0.902	PROTEASOME	0.924
ACTINY	0.902	RACCYCD	0.924
HIVNEF	0.902	AT1R	0.916
**BioCarta Network**	**Apparent Accuracy**	**BioCarta Network**	**Apparent Accuracy**
CELL2CELL	0.932	HIVNEF	0.955
P38MAPK	0.921	STRESS	0.944
G2	0.913	CHEMICAL	0.940
G1	0.911	DEATH	0.937
ERK	0.910	MCALPAIN	0.929
MPR	0.910	P38MAPK	0.926
PROTEASOME	0.908	G2	0.924
VEGF	0.905	IL2RB	0.924
CELLCYCLE	0.905	MPR	0.924
HIVNEF	0.904	CELLCYCLE	0.921

Top 10 networks for G2 vs. N (top left), G3 vs. N (top right), sGBM vs. N (bottom left), and pGBM vs. N (bottom right).

## Conclusions

We report here a systems approach to investigate molecular changes underlying astrocytoma pathology. Leveraging a large cohort of publicly available gene expression datasets, we have conducted the first meta-analysis that examines together the transcriptomes of three astrocytoma grades along with corresponding normal samples. We have combined individual gene- and network-based approaches to identify meaningful patterns of expression within and between different grades. The trend we observed of greater network dysregulation with higher grade represents a quantified measure of increasing inter-patient transcriptomic heterogeneity in more aggressive astrocytomas. We also identified genes that exhibit monotonically increasing or decreasing expression with increased grade–these genes are potentially reflective of shared oncogenic mechanisms among phenotypically similar tumors. Notably, monotonically increasing or decreasing changes in gene expression, parallel to increasing network dysregulation, presents a putative bridge between the known genetic heterogeneity of astrocytomas and expression heterogeneity at the population level, as analyzed in this meta-study.

Additionally, we identified networks distinguishing different astrocytoma grades from normal as well as network markers separating between glioma grades. This work presents significant results that enable better characterization of different human astrocytoma grades, and hopefully will lead to improvements in diagnosis and therapy choices.

## Supporting Information

Figure S1 **Pearson-correlation matrix before and after consensus pre-processing.** The heatmaps display correlation coefficients among all samples included in this study. The axes represent sample numbers. In the left figure, the purple borderlines of each box delineate different phenotypes, which coincide with the sample batches. Samples from the same laboratories or studies showed higher homogeneity than other samples. On the other hand, in the right figure, laboratory effects are much less obvious; tumor samples across different studies or phenotypes all look highly correlated with average correlation coefficient increased from 0.81 to 0.91.
(TIF)

Table S1 **Most differentially deregulated networks between different phenotypes.**
(CSV)

Table S2 **The chromosome locus and the putative functions of monotonically changing genes.**
(CSV)

Table S3 **Sensitivity, specificity, and accuracy using DIRAC to classify different grades of astrocytoma.**
(CSV)
