# Supplementary figures and images for: Transcriptional Analysis of Aggressiveness and Heterogeneity across Grades of Astrocytomas
Source: PLoS One. 2013 Oct 11;8(10):e76694. doi: 10.1371/journal.pone.0076694 (PMC3795736; doi:10.1371/journal.pone.0076694)

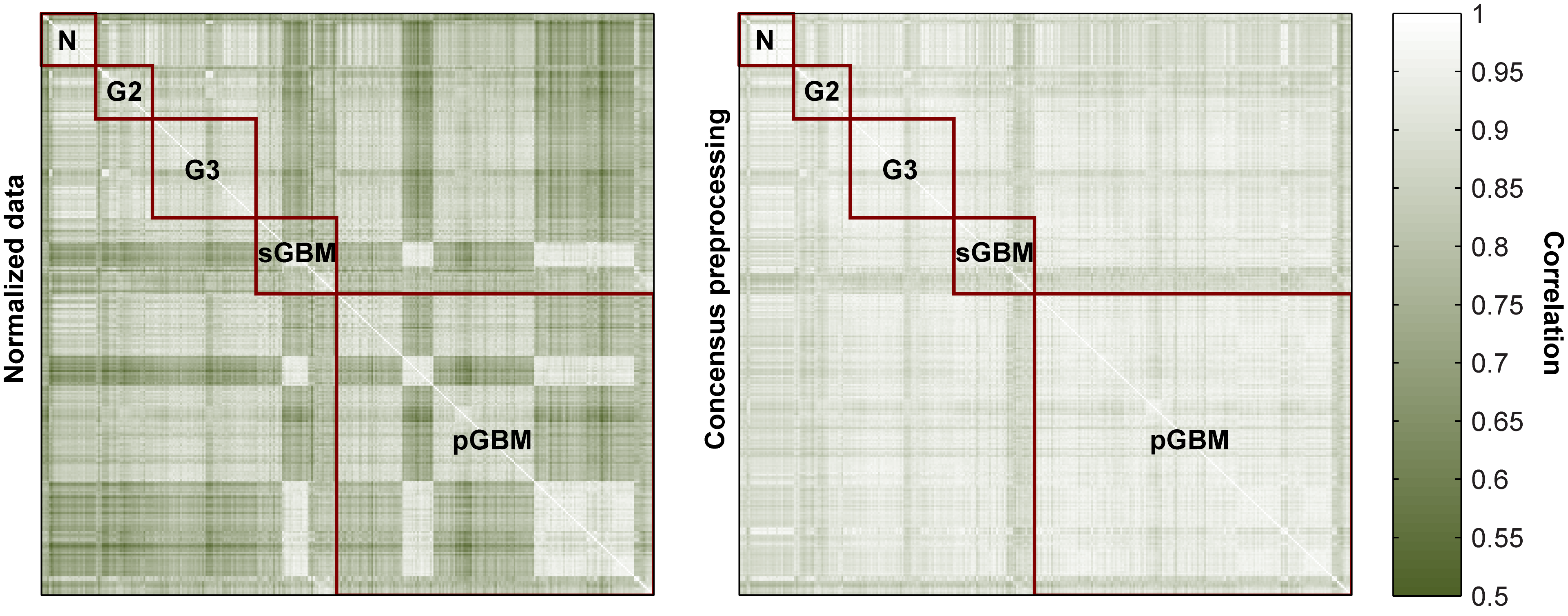

Supplement: Figure S1 — Pearson-correlation matrix before and after consensus pre-processing. The heatmaps display correlation coefficients among all samples included in this study. The axes represent sample numbers. In the left figure, the purple borderlines of each box delineate different phenotypes, which coincide with the sample batches. Samples from the same laboratories or studies showed higher homogeneity than other samples. On the other hand, in the right figure, laboratory effects are much less obvious; tumor samples across different studies or phenotypes all look highly correlated with average correlation coefficient increased from 0.81 to 0.91. (TIF) [file pone.0076694.s001.tif]
